# Effectiveness of medication in cluster headache

**DOI:** 10.1186/s12883-021-02195-8

**Published:** 2021-04-22

**Authors:** Johannes Drescher, Andreas Khouri, Tina Katharina Amann, Charly Gaul, Peter Kropp, Yannic Siebenhaar, Jörg Scheidt

**Affiliations:** 1 Institute of Information Systems, University of Applied Sciences Hof, Hof, Germany; 2 Institute of Medical Psychology and Medical Sociology, University of Rostock, Rostock, Germany; 3 Migraine and Headache Clinic Königstein, Königstein, Germany

**Keywords:** Cluster headache, Acute medication, Effectiveness, Citizen science

## Abstract

**Background:**

The aim of this work is to analyze the reports on cluster headache attacks collected online in the citizen science project CLUE with respect to the effectiveness of drugs taken during the attacks. The collection of data within the framework of citizen science projects opens up the possibility of investigating the effectiveness of acute medication on the basis of a large number of individual attacks instead of a simple survey of patients.

**Methods:**

Data from 8369 cluster headache attacks, containing information about acute medication taken and the assessment of its effect, were collected from 133 participants using an online platform and a smartphone app. Chi-square tests were used to investigate whether the effect of the three recommended acute drugs differs when distinguishing between participants with chronic or episodic cluster headache. Furthermore, it was investigated whether there are differences between smokers and non-smokers in the assessment of the effect of the acute medication.

**Results:**

Our participants rated the effectiveness of sumatriptan 6 mg s.c. as significantly better than oxygen and zolmitriptan nasal spray. Oxygen is considered to be significantly better in episodic versus chronic cluster headache, and sumatriptan is considered to be significantly better in chronic versus episodic cluster headache. Smokers rate the effect of oxygen as significantly better than non-smokers.

**Conclusions:**

Despite some methodological limitations, web-based data collection is able to support findings from clinical trials in a real world setting about effectiveness of acute cluster headache treatment in several situations.

**Supplementary Information:**

The online version contains supplementary material available at 10.1186/s12883-021-02195-8.

## Background

Patients suffering from cluster headaches experience the most intense pain of all primary headache disorders [1, 2].

Some randomized clinical trials investigated the effectiveness of different acute treatments. The effect of sumatriptan 6 mg s.c [3], .zolmitriptan 5 mg nasal spray [4] and the inhalation of pure oxygen [5] was demonstrated.

Further studies also distinguished between chronic and episodic cluster headache [6–8] and investigated whether there are differences between smokers and non-smokers [9]. This is of interest because there are significantly more smokers than nonsmokers among cluster headache patients than in the general population [10].

The purpose of this work is the analysis of cluster headache attack reports collected online in the citizen science project CLUE (https://cluster.kopfschmerz-radar.de/) with respect to the effect of drugs taken during the attacks. The CLUE project targeted cluster headache patients in German-speaking countries (Germany, Switzerland, Austria). No restriction on the age of the participants was given. The acquisition of participants was mainly done by communications of the cluster headache self-help groups to their members. The cluster headache self-help groups are organized in the Bundesverband der Clusterkopfschmerz-Selbsthilfe-Gruppen (CSG) e.V. (https://www.clusterkopf.de). This umbrella organization informed its members about the project.

This approach offers the possibility to verify those findings and their clinical impact in a real-world setting based on patients’ daily observations.

The collection of medical data via the web offers the possibility to collect a larger amount of data for a longer period of time in a larger region compared to other studies. However, we are aware of the shortcomings of this approach and will discuss these in detail.

## Methods

### The CLUE project

The CLUE project has been collecting data regarding cluster headache attacks using a web app as well as a smartphone app since March 2018. For data analysis, all data are anonymized. Participants can register in the project at any time and then start reporting their cluster headache. During the registration process, participants are informed about privacy issues. With their registration, they give their consent to participate.

The study was approved by the Ethics Committee of the Medical Faculty of the University of Rostock (reference number A 2017–0091).

### Study design

The purpose of the prospective study was to examine the effect of drugs taken during cluster headache attacks. The aim was to investigate whether there are differences in drug effectiveness between participants with chronic and episodic cluster headache. Furthermore, differences in effectiveness between smokers and non-smokers were to be investigated.

### Participants

Between March 21, 2018 and June 25, 2020, 315 German-speaking participants mainly from Austria, Germany and Switzerland registered in the project and reported their attacks. The reported attacks had to have occurred within the specified time period, they could be reported subsequently by July 5, 2020 at the latest. The diagnosis of cluster headache was performed using a headache questionnaire, which covers the diagnostic criteria of the International Headache Society (International Classification of Headache Disorders, ICHD-3 (beta version), 2). Participants with neither episodic nor chronic cluster headache were excluded. 282 participants remained in the sample. Table 1 shows the frequency with which the required symptoms were reported by these 282 participants.

**Table 1:** Symptoms according the ICHD-3 (beta) definition and their frequencies

Symptom	Frequency (***N*** = 282)
conjunctival injection and/or lacrimation	254 (90%)
nasal congestion and/or rhinorrhoea	267 (95%)
eyelid oedema	124 (44%)
forehead and facial sweating	139 (49%)
forehead and facial flushing	68 (24%)
sensation of fullness in the ear	124 (44%)
miosis and/or ptosis	186 (66%)

In addition, the Fagerström test was used to determine the nicotine dependence of smokers [11, 12]. We determined the length of the participation period in the project as the difference between the dates of the first and the last attack reported by the participant plus one. We included participants in the study if they participated for at least 10 days and reported at least 6 cluster headache attacks. In addition, participants who reported more than 25% of their attacks lasting longer than 180 min or equal 0 min were excluded.

For the analysis of the effectiveness of the drugs, a patient was only considered if he or she reported at least three attacks with the drug under consideration.

To compare the effectiveness of individual drugs, only attacks in which only one or two drugs were taken were considered. It was also requested that the dose taken should not be zero.

### Data collection

During the registration process, baseline characteristics were obtained including gender, year of birth, place of residence, occupational group (working (full-time, part-time), not working (pupil or student, retired, unemployed)) as well as shift work information (shift work: yes or no).

To record the individual cluster headache attacks, the participants entered information about the onset and end of the attack, the medication used and its dosage. The medication selection included sumatriptan, zolmitriptan, oxygen, lidocaine, ergotamine tartrate and ‘other’. The effectiveness of the drugs was recorded in three steps (yes, little, no). Other data, such as food and beverages consumed, which were not relevant for the present evaluation, were recorded for each attack.

For the analysis, individual profile data were linked to the data of each attack.

As mentioned above we use a web app and a smartphone app to record the attacks. The overall system is described in [13]. The recording mask is presented in the supplementary material to this publication. To assess the intensity of pain we use a numerical pain scale, which is described in [14].

### Statistics

The data were analyzed using the R language and the R-studio environment [15].

Chi-square tests were used to compare the distribution of the participants in the different groups like gender, cluster headache type (chronic, episodic) and the smoking and non-smoking groups.

Welch’s t-test was used to compare the age distributions of the several groups.

Chi-square tests were used to compare the effectiveness of drugs in different groups. The groups could represent different drugs (e.g. sumatriptan, oxygen), different cluster headaches (chronic, episodic) or the smoking and non-smoking groups. The effectiveness of the drugs was divided into two classes (“yes” and “little/no”) in accordance with [16]. The duration of participation and the number of reported attacks varied greatly among the participants. To ensure that individual participants with a large number of reported attacks did not dominate any result, the density distribution for each patient was included in the calculation of the Chi-square. The different number of attacks was then taken into account when calculating the statistical error of the components.

## Results

The final data set consisted of 13,649 cluster headache attacks of 139 participants who fulfilled the requirement of having reported at least 6 attacks within at least 10 days of participation. Of these, 133 participants (100 males; 33 females; ratio 3.0:1) also provided information about acute medication for 8369 attacks. This information included the drug taken, the dose and an assessment of its effectiveness.

Table 2 summarizes the characteristics of the participants.

**Table 2:** Patients’ characteristics

Characteristic	Patients^a^ (***N*** = 133)
Gender	
Male	100 (75%)
Female	33 (25%)
Age [years]	
Mean ± SD	42.3 ± 10.4
Range	22–68
Type of CH	
Episodic	98 (74%)
Chronic	35 (26%)
Smoker Information	
Smoker	78 (59%)
Non-Smoker	55 (41%)

*CH* Cluster headache

^a^Except for age

Table 3 divides the participants into smokers and non-smokers and into episodic and chronic cluster headache sufferers. The results of the statistical tests in the right-hand column show the balance of the groups in terms of gender distribution, age and cluster headache type.

**Table 3:** Division of participants into smokers and non-smokers and into episodic and chronic cluster headaches

	Patients^a^ (***N*** = 133)		
	**Smokers**	**Non-Smokers**	
**Frequency**	**78 (59%)**	**55 (41%)**	**Statistic**
**Gender** (male / female)	61 (78%) / 17 (22%)	39 (71%) / 16 (29%)	χ^2^ = 0.92 *p* = 0.34
**Age** [y], mean ± SD	41.3 ± 10.2	43.7 ± 10.8	*p* = 0.20
**CH – Type (**episodic / chronic)	57 (73%) / 21 (27%)	41 (75%) / 14 (25%)	χ^2^ = 0.04 *p* = 0.85
	**Episodic**	**Chronic**	
**Frequency**	**98 (74%)**	**35 (26%)**	**Statistic**
**Gender** (male / female)	74 (76%) / 24 (24%)	26 (74%) / 9 (26%)	χ^2^ = 0.02 *p* = 0.89
**Age** [y], mean ± SD	41.7 ± 10.8	43.7 ± 9.5	*p* = 0.31

^a^Except for age

**Fig. 1:**
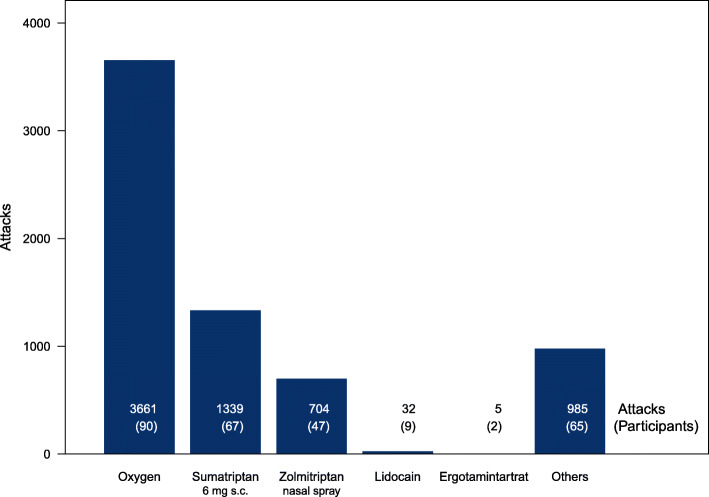
Distribution of the medications for attacks with only one medication reported

In 6726 of the 8369 attacks, the intake of only one drug was reported. The distribution of medication in these cases is shown in Fig. 1. In addition, the figure provides information on the number of participants reporting for each medication. Since each participant could report attacks with different medications, the sum of participants in Fig. 1 is greater than the total number of participants indicated above.

Due to the low case numbers, only the three most common medications, oxygen, sumatriptan 6 mg s.c. and zolmitriptan 5 mg nasal spray, were considered for the investigation of drug effectiveness. Although reporting oxygen flow was not mandatory, most participants (95.6%) reported plausible values between 4 and 20 l/min. The median was 13 l/min, the mode 15 l/min.

**Fig. 2:**
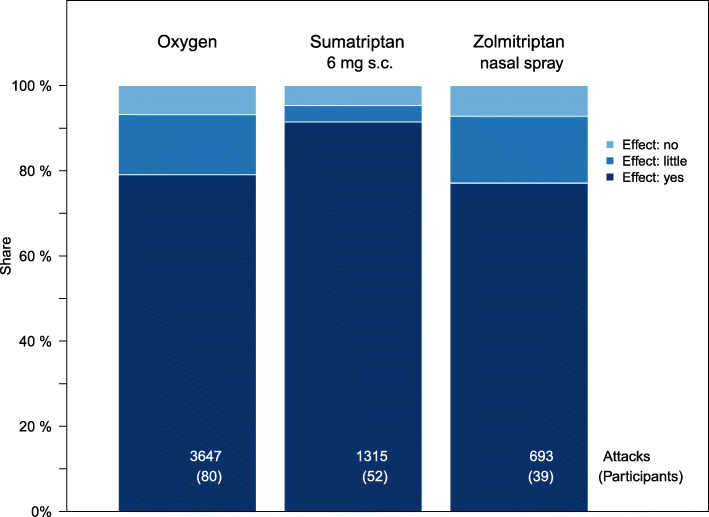
Effectiveness of the three medications under investigation

First, the effectiveness of all three drugs under consideration was examined and compared with each other. Figure 2 shows the three drugs with their effectiveness in the three gradations “yes”, “little” and “no”. As mentioned above, the effectiveness classes “little” and “no” were merged into one class for the investigation of the effects.

Comparing in pairs the assessment of the participants using sumatriptan or zolmitriptan or oxygen, their assessment of the effectiveness of the respective drug gives the following picture: The effect of sumatriptan 6 mg s.c. is estimated to be significantly better than that of oxygen, (*p* < 0.001, OR = 2.8), that of zolmitriptan 5 mg nasal spray not better than that of oxygen (*p* = 0.49) and the effect of sumatriptan 6 mg s.c. as significantly better than that of zolmitriptan 5 mg nasal spray (*p* < 0.001, OR = 3.2).

A comparison of the effect of the drugs by cluster headache type showed that oxygen is estimated to be significantly more effective for the treatment of episodic than of chronic cluster headache (*p* < 0.001, OR = 2.0). Sumatriptan 6 mg s.c. is estimated to be significantly more effective for the treatment of chronic than of episodic cluster headache (*p* = 0.03, OR = 2.2). For zolmitriptan 5 mg nasal spray (*p* = 0.39) there is no difference in the estimation of the effect between chronic and episodic cluster headache.

**Fig. 3:**
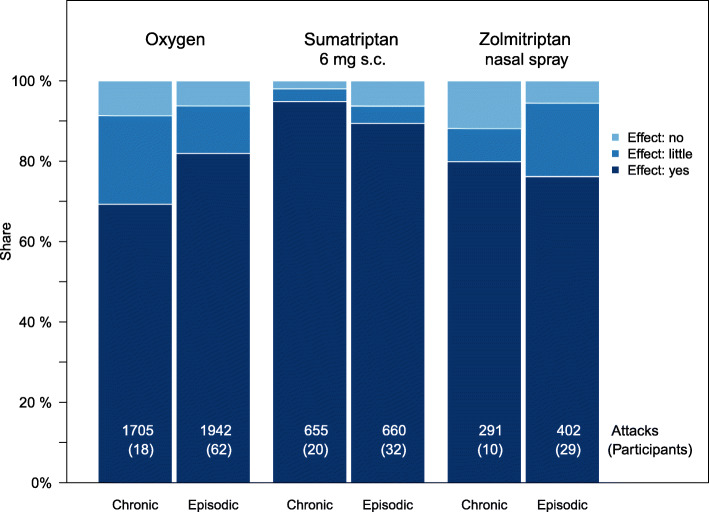
Effectiveness of the three medications under investigation by cluster headache type

Figure 3 shows the effectiveness of the drugs considered with the difference in headache type in chronic and episodic cluster headache.

The Fagerström test was used to investigate the nicotine dependence of our participants. The mean Fagerström score in our sample of smokers is 3.9. Twenty-seven of the smokers smoke more than 20 cigarettes per day and are therefore considered heavy smokers. This corresponds to a proportion of 35%.

**Fig. 4:**
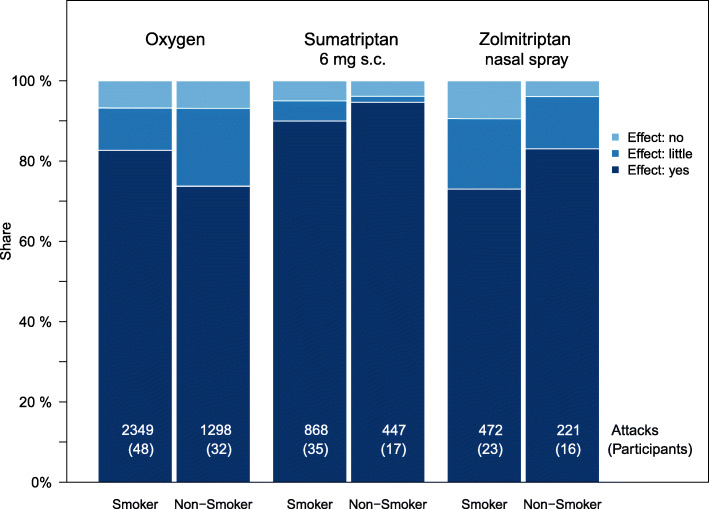
Effectiveness of the three medications under investigation by smoker and non-smoker

Comparing the drug effects between the smoking and non-smoking groups, the following picture emerged: Oxygen helps smokers significantly better than non-smokers (*p* = 0.001, OR = 1.7). Non-smokers rate the effect of triptans slightly better than smokers, although the differences are not or just as significant (sumatriptan 6 mg s.c.: *p* = 0.10, OR = 2.0; zolmitriptan 5 mg nasal spray: *p* = 0.05, OR = 1.8). Figure 4 shows the corresponding distributions.

Finally, we investigated whether the estimation of the drug effect improves when two of the drugs under consideration were taken. Twenty-five participants used both oxygen and sumatriptan 6 mg s.c. in 366 attacks, 18 participants took both oxygen and zolmitriptan 5 mg nasal spray in 331 attacks.

**Fig. 5:**
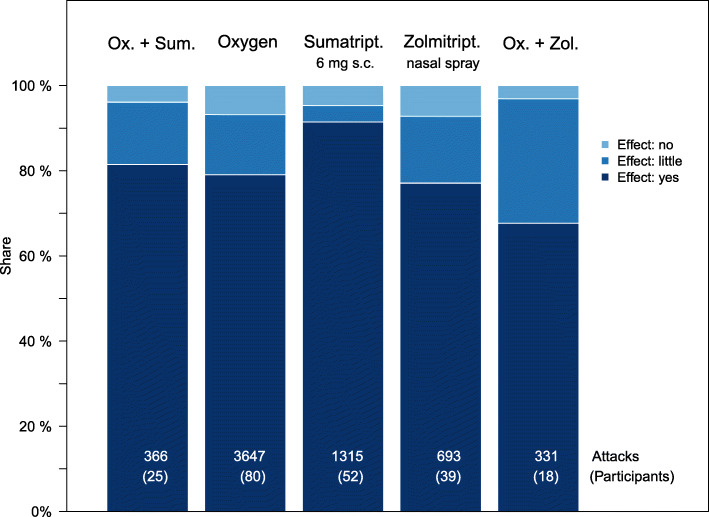
Effectiveness of combinations of the three medications under investigation

It was ascertained that the use of sumatriptan 6 mg s.c. in combination with oxygen does not improve the effectiveness (*p* = 0.43). The effectiveness of taking oxygen and zolmitriptan 5 mg nasal spray together is considered to be worse than taking only oxygen (*p* = 0.02, OR = 1.8) or only zolmitriptan (*p* = 0.08, OR = 1.6). Figure 5 shows the corresponding distributions.

## Discussion

We analyzed 13,649 cluster headache attacks from 139 patients collected in the citizen science project CLUE via a web app as well as a smartphone app. Diagnosis of cluster headache was based on ICHD-3 beta criteria (ICHD-3 beta, 2013). Compared to the more recent ICHD-3 (2018), the ICHD-3 beta includes “sensation of fullness in the ear” which was reported by 44% of the patients. The focus was on investigating the effectiveness of acute medication, with particular emphasis on distinguishing between the smoking and non-smoking groups and gender. Furthermore, the investigations differentiated between participants with episodic and chronic cluster headache. Since there were also three options for specifying medical effectiveness, we had to decide whether we wanted to combine “yes/little” or “little/no” to calculate an OR. In accordance with study [16], we decided to combine “little/no” as well.

The most commonly used drugs for the acute treatment of cluster attacks used were oxygen, sumatriptan 6 mg s.c. and zolmitriptan 5 mg nasal spray. Comparing these three in terms of effectiveness, it was found that sumatriptan 6 mg s.c. was reported as being significantly more effective than zolmitriptan 5 mg nasal spray and oxygen. The comparison of the latter two did not show any significant difference. These results are consistent with [3–5].

Differentiating the cluster headache types chronic and episodic in terms of the effect of the drugs, oxygen is significantly more effective in episodic than in chronic cluster headache. In the case of triptans, no difference can be observed in these two groups. These results confirm the study by Pearson et al. (2019) [16], but contradict two other studies. One study examined 124 patients (73% episodic and 27% chronic) and tested the effectiveness of oral zolmitriptan compared to placebos for both groups. Although they found a positive effect for episodic cluster headache, they noticed no effect in patients suffering from chronic cluster headache [6]. Another study found that subcutaneous sumatriptan was less effective for chronic cluster headaches than for episodic cluster headache [7].

Another result is the differentiation of drug effectiveness between the smoking and non-smoking groups. Here oxygen causes significantly better effects in smokers than in non-smokers. This is in accordance with [9], where differences between the effectiveness of sumatriptan and oxygen were studied. No significant differences in the effectiveness were revealed. However, when men and smokers were analyzed it was observed that their response to oxygen was significantly stronger.

An explanation might be the higher mean hemoglobin concentration in smokers compared to non-smokers which increases the oxygen delivery [9]. No such difference is found for triptans.

Our participants have a fairly high average Fagerström score of 3.9, and the proportion of heavy smokers is also high at 35%. Fagerström and Furberg calculated an average Fagerström score for smokers in Germany of 2.8 [17], and in a study by Lampert et al. the proportion of heavy smokers in Germany was reported to be 28% [18]. However, triggered by the non-smoking campaigns of recent years, the proportion of heavy smokers and thus also the average Fagerström score in Germany may have increased in recent years.

Another interesting result is the investigation of the combined intake of a triptan and oxygen. For both triptans, the effect of the triptan is not improved if oxygen is additionally applied.

However, we cannot say whether the drugs were taken together or whether some time elapsed between taking the first and second drug. In addition, we also do not know which of the two was used first.

A strength of the study is the data collection in a real world setting by apps, so the data and the conclusions are based on a large number of attacks of every participant, which is superior to obtaining data by a questionnaire. Also these data address real life while a questionnaire is based on perceptions which can be shifted retrospectively. On the other hand, participants could join or leave the study at any time. Even though a minimum participation period of 10 days was required, it cannot be guaranteed that participants actually reported all their attacks during this period. Selective reporting by the participants could also not be ruled out.

Furthermore, the diagnosis of cluster headache has not been confirmed by a physician. However, the questionnaire is based on the ICHD-3 (beta version) criteria and oxygen, nasal and subcutaneous triptans require prescription by a physician which indirectly confirms the diagnostic accuracy of our questionnaire.

Of course, the results on the effectiveness of acute treatments are not absolute, because the participants are taking the drugs they know will help them. We have no information about what acute treatments a participant had tried before and may have found less helpful. We also do not have information about when during an attack the medication was taken and how quickly it helped.

Furthermore, we have no information on prophylactic therapies. Therefore, our study cannot provide any information about their influence on the drug effectiveness.

## Conclusion

Overall, it can be said that the collection of study data within the framework of a citizen science project like CLUE can be an interesting addition to other clinical studies. This type of data collection allows nationwide data collection over a longer period of time with a large number of patients. Of course, in view of the weaknesses described above, the results obtained can only be interpreted with the necessary caution and should always be verified.

## Supplementary Information

**Additional file 1.** Supplementary Material: Online questionnaire to record the attacks.

## Data Availability

The datasets used and/or analyzed during the current study are available from the corresponding author on reasonable request.

## Abbreviations

ICHD: International Classification of Headache Disorders
IHS: International Headache Society
SD: Standard deviation
CLUE: Project ‘Clusterkopfschmerz erforschen”, funded by the German Federal Ministry of Education and Research (BMBF)
