# Supplementary material for: Effectiveness of medication in cluster headache
Source: BMC Neurol. 2021 Apr 22;21:174. doi: 10.1186/s12883-021-02195-8 (PMC8061078; doi:10.1186/s12883-021-02195-8)
Supplement: Supplementary file 1 — Additional file 1. Supplementary Material: Online questionnaire to record the attacks. [file 12883_2021_2195_MOESM1_ESM.docx]

# Effectiveness of Medication in Cluster Headache

Johannes Drescher^1, 2^, Andreas Khouri^1^, Tina Katharina Amann^1^, Charly Gaul^3^, Peter Kropp^2^, Yannic Siebenhaar^1^, Jörg Scheidt (corresponding author)^1^

1 - Institute of Information Systems, University of Applied Sciences Hof, Hof, Germany

2 - Institute of Medical Psychology and Medical Sociology, University of Rostock, Rostock, Germany

3 - Migraine and Headache Clinic Königstein, Königstein, Germany

## Supplementary Material: Online questionnaire to record the attacks

**Time and Place**

Begin of attack: Time: Date:

End of attack: Time: Date:

Place: Country: zip code: City:

**Medication**

Drug: Dosage:

Drug: Dosage:

Improvement through medication: □ yes □ no □ little

**Food**

Flavour enhancer □ yes □ no □ I don’t know

Histamin □ yes □ no □ I don’t know

Consumption of alcohol □ no □ yes, less than usual

□ yes, more than usual □ not specified

Type of alcohol □ Beer □ White wine □ Red wine

□ Hard liquor □ others

Consumption of caffeine □ no □ yes, less than usual □ yes, more than usual

Type of caffeine □ coffee □ tea □ energy drink □ others

**Pain Data**

Intensity < slider with scale from 0 to 10 >

Suspected trigger < free text field >

Personal remark < free text field >

**Other Information**

Unusual physical exertion □ yes □ no

Irritation of the nose □ yes □ no

Stimulus satiation □ yes □ no

Attack starts during sleep □ yes □ no

Sleep pattern □ normal sleep □ restless sleep
□ sleep too short □ sleep too long

Stressful situation □ no □ yes, attack before stressful situation

□ yes, attack during stressful situation □ yes, attack after stressful situation

**Menstruation**

Menstruation □ yes □ no □ not specified

Day of cycle < slider with scale from 0 to 7 >
